# Multidisciplinary Guidelines for the Rational Use of Topical Non-Steroidal Anti-Inflammatory Drugs for Musculoskeletal Pain (2022)

**DOI:** 10.3390/jcm12041544

**Published:** 2023-02-15

**Authors:** Chen Shi, Zhaoming Ye, Zengwu Shao, Bifa Fan, Cibo Huang, Yuan Zhang, Xinying Kuang, Liyan Miao, Xin’an Wu, Rongsheng Zhao, Xiao Chen, Bikui Zhang, Rongsheng Tong, Xin Hu, Zhijian Fu, Jianhao Lin, Xiaomei Li, Tiezheng Sun, Guoqiang Liu, Haibin Dai, Cheng Guo, Bo Zhang, Ting Xu, Aidong Wen, Xiaocong Zuo, Jinmei Liu, Xu Chen, Huibo Li, Jing Wang, Min Luo, Tingting Fan, Yulan Qian, Xiu’mao Li, Wenjie Qiu, Xiaowen Lin, Yingchang Pang, Yunfei Hou, Difei Yao, Wen Kou, Bao Sun, Can Hu, Yanzhe Xia, Ming Zhao, Changyu Zhu, Qian Li, Yu Zhang

**Affiliations:** 1 Department of Pharmacy, Union Hospital, Tongji Medical College, Huazhong University of Science and Technology (HUST), Wuhan 430022, China; 2 Hubei Province Clinical Research Center for Precision Medicine for Critical Illness, Wuhan 430022, China; 3 Department of Orthopedics, The Second Affiliated Hospital, School of Medicine, Zhejiang University, Hangzhou 310009, China; 4 Department of Orthopaedics, Union Hospital, Tongji Medical College, Huazhong University of Science and Technology (HUST), Wuhan 430022, China; 5 Department of Pain Management, China-Japan Friendship Hospital, Beijing 100029, China; 6 Department of Rheumatology, South China Hospital, Health Science Center, Shenzhen University, Shenzhen 518116, China; 7 Department of Health Research Methods, Evidence, and Impact, McMaster University, Hamilton, ON L8S 4K1, Canada; 8 Global Health Nursing, St. Luke’s International University, Tokyo 104-0044, Japan; 9 Department of Pharmacy, The First Affiliated Hospital of Soochow University, Suzhou 215006, China; 10 Department of Pharmacy, The First Hospital of Lanzhou University, Gansu 730000, China; 11 Department of Pharmacy, Peking University Third Hospital, Beijing 100191, China; 12 Institute for Drug Evaluation, Peking University Health Science Center, Beijing 100191, China; 13 Department of Pharmacy, The First Affiliated Hospital of Sun Yet-sen University, Guangzhou 510080, China; 14 Department of Pharmacy, The Second Xiangya Hospital, Central South University, Changsha 410011, China; 15 Department of Pharmacy, Sichuan Academy of Medical Sciences & Sichuan Provincial People’s Hospital, School of Medicine, University of Electronic Science and Technology of China, Chengdu 610072, China; 16 Personalized Drug Therapy Key Laboratory of Sichuan Province, Chengdu 610072, China; 17 Department of Pharmacy, Beijing Hospital, National Center of Gerontology, Institute of Geriatric Medicine, Chinese Academy of Medical Sciences, Beijing Key Laboratory of Drug Clinical Risk and Personalized Medication Evaluation, Beijing 100730, China; 18 Department of Pain Management, Shandong Provincial Hospital Affiliated to Shandong First Medical University, Jinan 250021, China; 19 Arthritis Clinic and Research Center, Peking University Peoples Hospital, 11 Xizhimen South Street, Xicheng District, Beijing 100044, China; 20 Department of Rheumatology and Immunology, The First Affiliated Hospital of USTC, University of Science and Technology of China, Lujiang Str. 17, Hefei 230001, China; 21 Department of Clinical Pharmacy, The Third Hospital of Hebei Medical University, Shijiazhuang 050051, China; 22 Department of Pharmacy, Second Affiliated Hospital of Zhejiang University School of Medicine, Research Center for Clinical Pharmacy, Zhejiang University, Hangzhou 310009, China; 23 Department of Pharmacy, Shanghai Sixth People’s Hospital Affiliated to Shanghai Jiao Tong University School of Medicine, Shanghai 200233, China; 24 Department of Pharmacy, State Key Laboratory of Complex Severe and Rare Diseases, Peking Union Medical College Hospital, Chinese Academy of Medical Sciences and Peking Union Medical College Hospital, Beijing 100730, China; 25 Department of Pharmacy, West China Hospital, Sichuan University, Chengdu 610041, China; 26 Department of Pharmacy, Xijing Hospital, Fourth Military Medical University, Xi’an 710032, China; 27 Department of Pharmacy, The Third Xiangya Hospital, Central South University, Changsha 410013, China

**Keywords:** topical non-steroidal anti-inflammatory drugs, musculoskeletal pain, pain management, guidelines

## Abstract

(1) Background: Topical non-steroidal anti-inflammatory drugs (NSAIDs) are one of the primary drugs for treating musculoskeletal pain. However, there are currently no evidence-based recommendations about drug selection, drug administration, drug interactions, and use in special populations or other pharmacology-related content of such medications. To this end, the Chinese Pharmaceutical Association Hospital Pharmacy Professional Committee developed multidisciplinary guidelines on using topical NSAIDs to treat musculoskeletal pain. (2) Methods: The guidelines development process followed the World Health Organization guideline development handbook, the GRADE methodology, and the statement of Reporting Items for Practice Guidelines in Healthcare. The guideline panel used the Delphi method to identify six clinical questions to be addressed in the guidelines. An independent systematic review team conducted a systematic search and integration of evidence. (3) Results: Based on the balance between the benefits and harms of an intervention, the quality of the evidence, patient preferences and values, and resource utilization, the guideline panel developed 11 recommendations and nine expert consensuses on using topical NSAIDs to treat acute and chronic musculoskeletal pain. (4) Conclusions: Based on the effectiveness and overall safety of topical NSAIDs, we recommend patients with musculoskeletal pain use topical NSAIDs and suggest high-risk patients use topical NSAIDs, such as those with other diseases or receiving other concurrent treatments. The evidenced-based guidelines on topical NSAIDs for musculoskeletal pain incorporated a pharmacist perspective. The guidelines have the potential to facilitate the rational use of topical NSAIDs. The guideline panel will monitor the relevant evidence and update the recommendations accordingly.

## 1. Introduction

Musculoskeletal diseases are one of the leading causes of disability globally. According to data in 2019, about 1.71 billion people worldwide have musculoskeletal disorders [1], and pain is the most common complaint. The prevalence of musculoskeletal pain in adults can range from 18% to 80% [2,3]. Musculoskeletal pain is also one of the leading causes of musculoskeletal disease burden [4]. According to the UK Health and Safety Executive (HSE), 5.7 million workdays were lost in 2001–2002 due to low back pain, and 4.1 million workdays were lost due to musculoskeletal disorders of the upper extremities and neck. Annual economic loss to individuals, industry, and society is appropriately 5.7 billion pounds [5]. Medical costs related to treating and caring for musculoskeletal diseases account for approximately 16.2% of all medical expenditures, creating a huge economic burden for society [6]. The burden of musculoskeletal disorders increases with population growth, urbanization, modern transportation, and increased longevity [7,8]. The years of living with disability (YLD) due to musculoskeletal disorders increased by 19.9% between 2000 and 2017 [4]. In China, YLD increased greatly from 17.6 million to 28.1 million from 1990 to 2019 [9]. Consequently, the management of musculoskeletal pain is essential.

Non-steroidal anti-inflammatory drugs (NSAIDs) are widely used for musculoskeletal pain. NSAIDs can block prostaglandin synthesis by inhibiting cyclooxygenase (COX) enzyme activity, thus alleviating pain and reducing inflammation associated with musculoskeletal diseases [10]. In the United States, 65.4% of osteoarthritis patients and 56.1% of patients with chronic low back pain are prescribed NSAIDs to treat pain [11]. In the United Kingdom, 10.36 million prescriptions for oral NSAIDs were written for musculoskeletal disorders in 2020 [12]. However, oral NSAIDs may not be appropriate in patients with higher risks of adverse events, such as gastrointestinal and cardiovascular events [13,14]. Topical NSAIDs provide effective analgesic concentrations only at the site of action. From a pharmacokinetic and pharmacodynamic perspective, topical application significantly reduces systemic drug exposure and may be safer than oral NSAIDs. Therefore, topical NSAIDs are increasingly used to treat acute and chronic musculoskeletal pain [15,16].

Although topical NSAIDs have been recommended in several management guidelines for musculoskeletal diseases, these guidelines [17,18,19,20] do not guide drug selection, administration, drug interactions, and use in special populations. Therefore, there is a research gap on these issues, and evidence-based guidelines are needed. The Chinese Pharmaceutical Association Hospital Pharmacy Professional Committee initiated a multidisciplinary guideline panel, inviting experts in pharmacy, clinical medicine, and evidence-based medicine to develop drug management guidelines for musculoskeletal pain based on the best available evidence. The guidelines aimed to promote a more standardized and rational use of topical NSAIDs in clinical practice and to ensure the effectiveness and safety of drug therapies.

## 2. Materials and Methods

### 2.1. The Guideline Scope, Target Professionals, and Target Patient Population

The guidelines apply to all levels of healthcare settings. The target patient population is those with acute and chronic musculoskeletal pain. The target healthcare professionals are clinicians (including orthopedics, pain specialists, rheumatologists, general practitioners, emergency physicians, plastic surgeons, rehabilitation physicians, physical therapists, sports physicians, and trauma surgeons), pharmacists, nurses, and other healthcare professionals and policymakers involved in musculoskeletal pain management.

### 2.2. The Methodology of the Guidelines Development

The detailed guideline development process has been published elsewhere [21]. Guidelines were registered on the International Practice Guidelines Registry Platform (PREPARE) (registration number IPGRP-2022CN265). The guideline development process and reporting followed the World Health Organization (WHO) handbook for guideline development [22], the GRADE methodology [23], and the Statement of Reporting Items for Practice Guidelines in Healthcare (RIGHT) [24]. Conflicts of interest and disclosure were managed under the policies of the International Committee of Medical Journal Editors (ICMJE).

### 2.3. Guidelines Panels

The guidelines panel comprised experts from the following disciplines: clinical pharmacy, pharmacoeconomics, pharmaceutics, orthopedic surgery, rheumatology and immunology, pain management, and guidelines development methodology. Members were divided into a steering group, a development group, a secretary group, and an external review group according to their responsibilities. The member composition and responsibility details are shown in Supplemental Table S1.

### 2.4. Clinical Questions and Outcome Index Construction

Preliminary clinical questions were proposed according to the PICO principles: P (Patient), I (Intervention), C (Control), and O (Outcome). The steering group selected the initial clinical questions and outcomes based on relevant literature, their clinical experience, and communication with other healthcare providers. Subsequently, the development group assessed the importance of research questions (1 = not important, and 5 = critical) and outcomes (1 = not important at all, and 9 = very important) using Likert-like scales through three rounds of Delphi questionnaire consultation [17,18,19,20]. Finally, the team determined six clinical questions and 23 outcomes (Supplemental Table S2).

### 2.5. Evidence Retrieval, Data Extraction, and Methodological Quality Assessment

An independent systematic review group completed systematic reviews for all PICO questions [25]. The selection of eligible studies followed PICO information in Supplement Table S2. We included systematic reviews, meta-analyses, and randomized controlled trials (RCT) on efficacy and safety outcomes. However, when RCT evidence is not available, we included non-randomized studies. We searched six English and Chinese electronic databases, including Medline, Embase, Cochrane Database of Systematic Reviews (CDSR), China National Knowledge Infrastructure (CNKI), Wanfang Database, and China Biomedical Database (CBM), from inception to July 2022. Manual searches were performed on references, gray literature, and studies of pharmacokinetics, pharmacodynamics, and pharmacoeconomics.

Two reviewers independently screened the title, abstract, and full-text records for potentially relevant studies and then extracted data from included studies using a standardized data extraction form. Two reviewers cross-checked the screening results and data extraction forms, and disagreements were resolved through group discussions led by another senior researcher. After the evidence retrieval was completed, the corresponding assessment tool [26,27,28,29,30,31,32] was selected according to the type of study to assess the risk of bias in each study (Supplemental Table S3). Two methodologists independently carried out the quality assessment. When there was a dispute about the bias assessment of the study, e.g., selection bias, detection bias, attrition bias, etc., it was resolved through group discussions led by another senior researcher.

### 2.6. Comprehensive Analysis of Evidence and the Evidence Report Preparation

A systematic review was adopted when it was relevant and of high quality (representing the PICO questions, published in the last two years, and evaluated as high quality by AMSTAR 2 [27]). Otherwise, the systematic review was updated to include more recent evidence, or a new systematic review was performed. Meta-analyses were performed by updating two Cochrane systematic reviews for clinical question 1. These two reviews used a fixed-effect model to estimate the pooled effects for categorical outcomes, including clinical success and adverse events, as there was no significant heterogeneity between the studies. The pairwise comparisons of topical NSAIDs versus placebo or oral NSAIDs were updated with additional eligible studies using RevMan 5.3. For other research questions, we could not conduct meta-analyses due to heterogeneity in participants, interventions, outcomes, statistics of the included studies, or a limited number of studies. The results of the included studies were tabulated by outcomes. Categorical (serious adverse events) or continuous outcomes (pain score), as listed in Supplement Table S2, were included. The GRADE (Grading of Recommendations, Assessment, Development, and Evaluation) standard was used to grade the quality of evidence for each clinical question [33]. The quality of the evidence was divided into high (A), moderate (B), low (C), and very low (D) ( Supplemental Table S4). If insufficient evidence was found for a question, expert evidence (E) was collected to supplement the evidence [34]. Finally, the team developed an evidence summary table, which was reviewed by the methodologists and at the consensus meeting.

### 2.7. Assessing Patient’s Values and Preferences

The guideline panel conducted a questionnaire-based survey to assess patients’ values and preferences in pain management. The questionnaire included questions about the effectiveness, safety, and prognosis of treatment. We recruited 318 patients (or caregivers) with musculoskeletal pain. The results of this survey were submitted to the guideline panel to provide references to make recommendations.

### 2.8. The Process of Formulating Recommendations

For each clinical question, the development group proposed recommendations based on the balance of benefits and harms suggested by the best available evidence, evidence quality, patient values and preferences, cost and cost-effectiveness, feasibility, and acceptability. The levels of recommendations are a “strong recommendation (recommend … for or against use)” and a “weak recommendation (suggest … for or against use)” based on the GRADE recommendation table [35,36]. When a specific clinical question lacked direct and supporting evidence, the development group collected expert clinical experience through an online questionnaire. The group then created the first draft of the expert consensus after a discussion based on expert evidence. (Table 1). Finally, we sought feedback from the guideline panel for the wording of recommendations and expert consensus statements through an anonymous online questionnaire survey. The consensus for language was reached when at least 3/4 of the voting experts agreed.

### 2.9. External Review of the Guidelines

After the guideline panel drafted the guidelines following the RIGHT checklist [24], the guidelines were peer-reviewed by external journal reviewers. Then, the guideline panel modified the draft guidelines based on the feedback and finalized the full guidelines. Finally, the guidelines were approved by the Chinese Pharmaceutical Association Hospital Pharmacy Professional Committee for publication and distribution.

## 3. Results

### 3.1. The Characteristics of Topical NSAIDs

The dosage forms of topical NSAIDs include, but are not limited to, solutions, gels, creams, ointments, sprays, aerosols, cataplasms, and patches. Topical preparations usually contain three components: active ingredients, excipients, and transdermal absorption enhancers. These components significantly influence the preparation’s characteristics and clinical features [37].

An ideal topical preparation should have a low molecular weight (<500 Da), be potent, and be both hydrophilic and hydrophobic [38]. Unfortunately, no NSAIDs have all these properties. Topical NSAIDs usually need to add transdermal absorption enhancers to increase absorption. In addition to these enhancers, some components of excipients, such as ethanol and glycerin, improve skin permeability. However, these additives can also irritate the skin. Therefore, topical preparations are generally contraindicated for incomplete or rash-affected skin, eyes, and other mucous membranes. Furthermore, ethanol and propylene glycol are common allergens, and attention should be paid to the patient’s allergy history.

### 3.2. Marketed Topical NSAIDs

Various topical NSAIDs have been approved worldwide, including ten dosage forms and more than ten pharmaceutical ingredients (Supplemental Table S5). Indications for these products include local inflammation and pain caused by various reasons. The usual method of administration is a direct application to the pain site. Non-patch topical NSAIDs can also be patted or massaged to facilitate drug absorption into the skin. Existing studies have shown that after topical administration, the drug concentration of subcutaneous fat, tendon, muscle, and periosteum of local tissue is 2.05–6.61 times that of oral administration [39,40,41]. The maximum plasma topical drug concentration (Cmax) is less than 10% of the oral dosage form [40,41,42,43]. The time to reach the maximum concentration (Tmax) is also approximately ten times that of the oral dosage form, and the bioavailability is 1–7% of the oral administration (except etofenamate, which achieves 21% bioavailability) [40,41,43,44]. These pharmacokinetic properties indicate that topical NSAIDs have minimal and slow systemic absorption and exert action in local tissues. The penetration kinetics of different topical NSAIDs may vary significantly due to the choice of excipients and transdermal absorption enhancers. Even between products with the same active ingredient, significant differences may exist due to skin pharmacokinetics and application site variability, which may substantially impact the formulation’s clinical efficacy. Therefore, different topical formulations of the same drug are not pharmaceutically or clinically interchangeable [45]. When selecting topical preparations, it is necessary to comprehensively assess the patient’s characteristics, treatment adherence, drug components, and drug delivery systems.

Due to the low systemic absorption, compared to oral formulations, the incidence of systemic adverse events of topical NSAIDs is very low. Adverse events are mainly local skin irritation or reaction at the application site, manifested as redness, rash, and itching [46,47]. These adverse events quickly subside after drug discontinuation. In general, topical NSAIDs are safer than oral formulations. However, there may be allergy-like reactions that are not immunologically mediated between NSAIDs but rather due to the loss of mast cell-stabilizing prostaglandins (PGE2) and the excessive production of cysteine leukotrienes as a result of the inhibition of COX-1. Such reactions are known to be cross-reactive, with patients reacting to NSAIDs from chemically unrelated groups (cross-reactive hypersensitivity) [48]. Thus, cross-allergy may be present between the two formulations. Patients who are allergic to oral formulations should be cautioned against using topical NSAIDs.

### 3.3. Evidence Summary and Recommendations

#### 3.3.1. Clinical Question # 1. Should Patients with Musculoskeletal Pain Use Topical NSAIDs for Pain Relief?

##### Acute Musculoskeletal Pain

A Cochrane systematic review on topical NSAIDs for acute musculoskeletal pain published in 2015 was included [15]. This review was updated with an additional 18 studies: 11 compared to placebo [49,50,51,52,53,54,55,56,57,58,59], one compared to oral NSAIDs [60], three compared to topical NSAIDs [61,62,63], and three compared to other topical drugs [64,65,66].

(1) compared to the placebo

The treatment success of topical NSAIDs and the risk of local adverse events were compared to the placebo. Topical NSAIDs included diclofenac, ibuprofen, ketoprofen, piroxicam, indomethacin, benzydamine, and etofenamate. The use of topical NSAIDs in patients with acute musculoskeletal pain had a higher rate of treatment success (pain relief) compared to placebo (relative risk (RR) 1.55, 95% confidence interval (95% CI) 1.48–1.64, *p* < 0.00001). The number needed to treat (NNT) was 5 (95% CI 3.67–4.89). Compared to placebo, topical NSAIDs did not increase the risk of local adverse events (RR 1.01, 95% CI 0.84–1.21, *p* = 0.93) ( Supplemental Table S6).

(2) compared to oral NSAIDs

Three RCTs found that topical and oral NSAIDs (indomethacin, ibuprofen, loxoprofen) had similar treatment success rates [60,67,68]. The risk of local adverse events was similar between the two formulations. However, topical NSAIDs had a lower risk of systemic adverse events (2% vs. 8%) [15] and gastrointestinal adverse events (0.8% vs. 12.8%) [60].

(3) compared to other topical medications

Four RCTs compared topical NSAIDs and other topical medications for acute musculoskeletal pain [64,65,66,69]. Due to the significant comparison heterogeneity among the included studies, data synthesis could not be performed.

##### Chronic Musculoskeletal Pain

A 2016 Cochrane systematic review on topical NSAIDs for chronic musculoskeletal pain was included [16]. This review was updated with an additional 42 studies: 14 compared to placebo [70,71,72,73,74,75,76,77,78,79,80,81,82,83], four compared to oral NSAIDs [84,85,86,87], five compared to topical NSAIDs [88,89,90,91,92], and 19 compared to other topical drugs [93,94,95,96,97,98,99,100,101,102,103,104,105,106,107,108,109,110,111].

(1) compared to the placebo

Topical diclofenac had a higher rate of treatment success (pain relief) than placebo (RR 1.66, 95% CI 1.38–2.00, *p* < 0.00001) when taken for two to six weeks for chronic musculoskeletal pain. When taken for more than six weeks, a high treatment success rate was maintained (RR 1.20, 95% CI 1.12–1.29, *p* < 0.00001). The NNT was 11 (95% CI 7.56–18.27). Differences in local adverse event risks were not statistically significant (RR 1.13, 95% CI 1.00–1.29, *p* = 0.06), and so was the difference for gastrointestinal adverse events (RR 1.13, 95% CI 0.82–1.55, *p* = 0.46). However, topical diclofenac had a higher risk of discontinuation due to adverse events (RR 1.46, 95% CI 1.10 to 1.94, *p* = 0.009) ( Supplemental Table S7). Compared to placebo, topical ketoprofen for chronic musculoskeletal pain had a higher rate of treatment success (RR 1.09, 95% CI 1.01–1.17, *p* = 0.03). The NNT was 25 (95% CI 12.90–219.27). The risks of local adverse events (RR 1.04, 95% CI 0.85–1.27, *p* = 0.71), gastrointestinal adverse events (RR 0.96, 95% CI 0.69–1.32, *p* = 0.79), and drug discontinuation (RR 1.28, 95% CI 0.92 to 1.78, *p* = 0.14) for ketoprofen were comparable to those of placebo (Supplemental Table S7).

(2) compared to oral NSAIDs

In addition, we evaluated the efficacy and safety of topical and oral NSAIDs in patients with chronic musculoskeletal pain (Supplemental Table S8). The probability of successful pain relief with topical NSAIDs was comparable to that with oral NSAIDs (RR 1.04, 95% CI 0.97–1.12, *p* = 0.26). However, topical NSAIDs had a higher risk of treatment discontinuation due to poor efficacy (RR 1.98, 95% CI 1.25–3.12, *p* = 0.003) and a higher risk of local adverse events (RR 3.36, 95% CI 2.53–4.45, *p* < 0.00001). Compared to oral NSAIDs, topical NSAIDs had better gastrointestinal safety (RR 0.66, 95% CI 0.56–0.77, *p* < 0.00001). The risk of treatment discontinuation due to adverse events was comparable between topical and oral NSAIDs (RR 0.84, 95% CI 0.68–1.05, *p* = 0.13).

(3) compared to other topical medications

Twenty-two RCTs compared the efficacy and safety of topical NSAIDs with complementary and alternative topical medications or capsaicin creams to treat chronic musculoskeletal pain. Due to the significant comparative heterogeneity among the included studies, data synthesis could not be performed.

##### Other Considerations

In surveys, patients with musculoskeletal pain preferred topical NSAIDs, with an average willingness score of 4.23 or more (1 = not willing at all; 5 = very willing) (Supplemental Figure S1). More patients were willing to choose topical NSAIDs than oral NSAIDs (47.70% vs. 17.57%) (Supplemental Figure S2).

Rationale: In conclusion, our evidence review found that topical NSAIDs are superior to placebo. While comparing topical versus oral NSAIDs, topical NSAIDs lead to comparable pain relief, and higher risk of local adverse events, but they are generally safer in systemic adverse events. In addition, patients had preferences for topical administration over oral administration. Therefore, the guideline panel made the following recommendations for using topical NSAIDs.

Recommendation 1: Compared to no treatment, given the safety and efficacy of topical NSAIDs, we recommend that patients use topical NSAIDs for acute musculoskeletal pain (1A).

Recommendation 2: Topical NSAIDs are as effective as oral NSAIDs in treating acute musculoskeletal pain but with greater safety. We suggest patients choose topical or oral NSAIDs for acute musculoskeletal pain (2D).

Recommendation 3: Compared to no treatment, given the safety and efficacy of topical NSAIDs, we recommend that patients use topical NSAIDs for chronic musculoskeletal pain (1B).

Recommendation 4: The efficacy of topical NSAIDs in treating chronic musculoskeletal pain is similar to oral NSAIDs but with greater safety. We suggest patients choose topical or oral NSAIDs for chronic musculoskeletal pain (2B).

#### 3.3.2. Clinical Questions #2. What Topical NSAIDs Should Be Recommended to Relieve Pain in Patients with Musculoskeletal Pain?

Two high-quality Cochrane systematic reviews [15,16], one Cochrane review [112], and a network meta-analysis published in 2021 [113] were included. Subgroup analyses were performed on the treatment success rates of different topical NSAIDs according to drug ingredients and dosage forms.

Compared to placebo, the following topical NSAIDs with different gradients all had higher treatment success rates: diclofenac (RR 1.55, 95% CI 1.44–1.66), ibuprofen (RR 1.84, 95% CI 1.58–2.14), ketoprofen (RR 1.56, 95% CI 1.37–1.77), piroxicam (RR 1.48, 95% CI 1.27–1.73), indomethacin (RR 1.26, 95% CI 1.03–1.55), benzydamine (RR 1.15, 95% CI 0.96–1.38, and etofenamate (RR 6.83, 95% CI 3.08–15.16) (Supplemental Table S9).

Among the different topical diclofenac formulations, the gel formulation had the highest treatment success rate (RR 3.84, 95% CI 2.68–5.50, *p* < 0.00001). The therapeutic effects of the ibuprofen gel (RR 2.66, 95% CI 1.69–4.21, *p* < 0.0001) and plaster (RR 2.13, 95% CI 1.70–2.68, *p* < 0.00001) were better than those of the cream formulation (RR 1.28, 95% CI 1.03–1.59, *p* = 0.03). Among the ketoprofen formulations, the gel formulation was relatively effective (RR 2.19, 95% CI: 1.74–2.75, *p* < 0.00001).

Furthermore, a network meta-analysis analyzed the efficacy and safety of NSAIDs and other drug treatments for knee and hip osteoarthritis [113]. Ninety drugs or doses were compared: 68 NSAIDs, 19 opioids, and 3 acetaminophen treatments. Topical diclofenac (70–81 and 140–160 mg/day) and flurbiprofen patch (40 mg) had effect sizes of pain relief greater than 0.5 and 0.3, respectively. These topical NSAIDs achieved more than a 50% probability of attaining minimal clinically relevant pain reduction. Although the difference was not statistically significant, topical diclofenac (140–160 mg/day) had the highest risk of discontinuation due to adverse events (RR 1.58, 95% CI: 0.77–3.34), and flurbiprofen patch (40 mg) had the lowest risk of discontinuation (RR 0.32, 95% CI: 0.01–3.82). Therefore, topical diclofenac was the most effective treatment but had a higher risk of discontinuation. The flurbiprofen patch (40 mg) had good pain relief and a relatively better safety profile (Supplemental Table S10).

A meta-analysis was performed to compare piroxicam and indomethacin based on the evidence in Clinical Question 1. Topical piroxicam had greater treatment success than topical indomethacin (RR 1.2, 95% CI 1.1–1.4). The flurbiprofen cataplasm provided better pain relief than the indomethacin cataplasm in treating acute and chronic orthopedic pain in an RCT [114].

Among the different dosage forms, patients with musculoskeletal pain preferred cataplasms and patches, with an average of >4 points (relatively willing), which was followed by sprays and gel. Creams were the least preferred (3.64 points) (Supplemental Figure S3).

Rationale: In conclusion, the quality of evidence comparing different topical NSAIDs was low or very low. The guideline panel found that the evidence was not sufficient to support recommendations. However, with limited evidence and patient preference considerations, the panel made the following statements on the rational use of topical NSAIDs.

Expert Consensus 5: When topical NSAIDs are used for musculoskeletal pain, topical NSAIDs with different ingredients have a relatively high possibility of treatment success compared to placebo. There is relatively sufficient evidence to support the use of diclofenac, flurbiprofen, ibuprofen, ketoprofen, and piroxicam (3C).

Expert Consensus 6: When topical NSAIDs are used for musculoskeletal pain, the quality of evidence on the therapeutic effects of different formulations of topical NSAIDs is very low. Physicians and patients are suggested to choose appropriate dosage forms based on the underlying diseases, the etiology and location of pain, and individual preferences (3C).

#### 3.3.3. Clinical Question #3. Should Fixed-Dose or on-Demand Topical NSAIDs Be Used for Patients with Musculoskeletal Pain?

After comprehensive evidence collection and screening, no RCTs or non-RCTs met the inclusion criteria. To form guiding recommendations and standardize clinical practice, expert clinical experience on the fixed-dose or on-demand use of topical NSAIDs was collected through an online questionnaire. The survey found that the composition and dosage forms used in these therapies were not fixed in clinical practice. On-demand treatment depends on the pain area or pain relief from the previous administration. Fixed-dose treatment generally followed the drug instructions with the scheduled number of daily uses and duration of therapy and was typically for patients with chronic musculoskeletal pain. If tissue damage was minor, the therapeutic effect between the two modalities was not significantly different. No significant differences in safety were observed between the two methods.

Rationale: In conclusion, there was no direct applicable evidence for comparing the use of fixed-dose or on-demand topical NSAIDs. The guideline panel made the following statements based on expert evidence to guide the rational use of topical NSAIDs.

Expert Consensus 7: Clinicians or pharmacists can choose fixed-dose or on-demand topical NSAIDs for musculoskeletal pain according to pain severity, etiology, patient’s conditions and preferences, and therapeutic response to topical NSAIDs. Fixed-dose therapy may be more advantageous in patients with chronic polyarticular musculoskeletal pain or combined muscle and joint pain. Medication orders for fixed-dose topical NSAIDs can follow drug instructions. The fixed dose and duration of use are convenient for patients to adhere to the medication order (3E).

#### 3.3.4. Clinical Question #4. When Patients with Musculoskeletal Pain Use Topical NSAIDs, Should Clinicians Be Concerned about Drug Interactions with Oral NSAIDs, Oral Acetaminophen, Warfarin, Angiotensin-Converting Enzyme Inhibitors (ACEI), Angiotensin-II Receptor Blockers (ARB), or β-Blockers

A post hoc RCT analysis analyzed the safety of topical diclofenac and other drug interactions. The risk of adverse events was 62.6% in patients taking other drugs known to interact with NSAIDs (n = 171). In contrast, the risk of adverse events was 55.4% in patients who did not use medications known to interact with NSAIDs (n = 83) (Supplemental Table S11). These results indicate that topical diclofenac, in combination with other drugs, did not increase the risk of adverse events [115].

##### Combination with Oral NSAIDs

The efficacy and safety between oral and topical diclofenac combination therapy and topical or oral diclofenac monotherapies were compared in an RCT [116]. There were no significant differences between combination therapy and monotherapy in pain relief, physical function, overall health assessment, and global assessment. The risk of adverse events was similar between these groups: 64.5% in the combination therapy group, 62.3% in the topical diclofenac group, and 62.3% in the oral diclofenac group. However, combined flurbiprofen cataplasm therapies with meloxicam tablets or etofenamate gel with sustained-release capsules of ibuprofen effectively reduced the pain level of osteoarthritis patients without increasing the occurrence of adverse events compared to oral NSAID monotherapy [117,118]. In an open-label, parallel-group, multicenter trial, a half dose of oral NSAIDs combined with feldene gel, compared to oral NSAIDs alone, had similar symptom scores in osteoarthritis patients and was well tolerated. However, the combined therapy group benefited more regarding tenderness, activity limitation, and quality of life assessment [119].

##### Combination with Oral Acetaminophen

An RCT compared ketoprofen patches combined with acetaminophen and acetaminophen monotherapy in patients with osteoarthritis. More significant analgesia was observed in the combination group [120]. Meanwhile, of the 65 clinical trials on topical NSAIDs, 57% allowed patients to use acetaminophen as rescue therapy [120]. Furthermore, a supplementary data analysis summarizing four RCTs found that the concurrent use of oral acetaminophen and oral ibuprofen did not increase the risk of common adverse events [121].

##### Combination with Warfarin

Five adverse events with an enhanced anticoagulant effect of warfarin were reported in the literature, one of which resulted in gastrointestinal bleeding [122] (Supplemental Table S11). Since the number of cases of combined uses of topical NSAIDs and warfarin was unknown, it is impossible to determine whether the combination therapy increased the risk of adverse events of warfarin.

##### Combination with ACEI, ARB, or β-Blockers

There was no evidence for the combined use of ACEI, ARB, or β-receptor blockers with topical NSAIDs. Drug metabolism studies of oral and topical NSAIDs suggest that compared to oral administration, topical NSAIDs had lower blood drug concentrations and less impact on organ systems. Taburet et al. studied the drug metabolism of topical and oral flurbiprofen in healthy subjects. They found that topical flurbiprofen had a lower Cmax and a more delayed Tmax than oral flurbiprofen [41].

Rationale: In conclusion, there was limited evidence for using topical NSAIDs in combination with other medications. Considering the metabolism characteristics, topical NSAIDs may be safer than oral NSAIDs. The guideline panel made recommendations on the combined use of topical NSAIDs with other drugs based on limited evidence and the pharmacokinetic characteristics of topical NSAIDs.

Recommendation 8: We suggest that topical or oral NSAIDs be used alone to treat musculoskeletal pain. We suggest topical NSAIDs as the first-line choice for patients with mild musculoskeletal pain. If symptoms persist or for moderate to severe pain, oral NSAIDs or analgesics with other mechanisms of action can be used after a comprehensive evaluation (2D).

Recommendation 9: We suggest oral acetaminophen as short-term rescue therapy if necessary when using topical NSAIDs in patients with musculoskeletal pain (2D).

Recommendation 10: There are case reports that topical NSAIDs can enhance the anticoagulant effect of warfarin. We suggest that patients with musculoskeletal pain monitor the international normalized ratio (INR) value when using a combination of topical NSAIDs and warfarin (2D).

Expert consensus 11: There is no evidence that the combination of topical NSAIDs and ACEI, ARB, β-blockers, or other cardiovascular drugs will increase the risk of adverse events.

#### 3.3.5. Clinical Question #5. Can Patient Populations with a High Risk of Adverse Events Use Topical NSAIDs?

##### Pregnant Patients

For pregnant patients, there is currently no evidence to evaluate the efficacy and safety of topical NSAIDs in the treatment of musculoskeletal pain. The US FDA recommends avoiding NSAIDs after 20 weeks of pregnancy to prevent fetal kidney damage [123]. However, this recommendation does not explicitly specify whether it applies to topical NSAIDs. A systematic review evaluated the safety risks of NSAIDs in the second trimester [124]. Of the 681 included studies, 26 had relevant information on adverse effects. Premature labor was the main reason for second-trimester indomethacin treatment, while other clinical indications for NSAIDs (e.g., musculoskeletal diseases) were under-represented. The narrowing or closure of the ductus arteriosus in the second trimester was described in 33 fetuses. Only eight studies reported adverse effects after less than seven days of exposure to NSAIDs during the second trimester. The short-term use of NSAIDs as analgesics or antipyretics in the second trimester did not appear to pose a substantial risk of fetal adverse effects.

##### Lactating Patients

Currently, there is no evidence to evaluate the efficacy and safety of topical NSAIDs in treating musculoskeletal pain in lactating patients. Studies have shown that except for aspirin, most NSAIDs are less excreted in breast milk after oral administration [125]. Therefore, COX-1 and COX-2 inhibitors are generally considered safe and preferable to aspirin when breastfeeding [125]. LactMed, a drug and lactation database of the National Institutes of Health (NIH), states that ibuprofen has an extremely low content in breast milk with a short half-life. The safe dose of ibuprofen in infants is much higher than the level of the drug excreted in breast milk. Therefore, ibuprofen is the preferred analgesic or anti-inflammatory drug for lactating women [126].

##### Patients with Hepatic Insufficiency

There is no evidence to evaluate the efficacy and safety of topical NSAIDs in patients with liver disease or liver dysfunction. NSAIDs have a low risk of liver injury, with an incidence rate of 0.29–9/100,000. Nimesulide and diclofenac have a relatively high risk of liver injury [127,128]. The increased risk of liver injury caused by diclofenac may be associated with higher doses (150 mg or more) and long-term treatments (over 90 days) [129]. Therefore, appropriate drugs and doses should be selected according to the patient’s condition and the severity of liver dysfunction [130].

##### Patients with Renal Insufficiency

Although there is indirect evidence comparing kidney injury in patients with osteoarthritis and back pain treated with topical and oral NSAIDs in the Japanese National Health Insurance database, topical NSAIDs had a similar risk of kidney injury compared to oral NSAIDs [131]. No serious renal adverse events have been reported with topical NSAIDs [132]. High doses of NSAIDs are associated with acute kidney injury (AKI), and the long-term use of NSAIDs increases the risk of chronic kidney disease (CKD). When NSAIDs must be used, clinicians should carefully select the appropriate drug and dose [133].

##### Pediatric Patients

Topical NSAIDs can be safe and effective in pediatric patients. An RCT of 112 children with acute sprains found that ketoprofen gel provided short-term pain relief without an increased risk of adverse events [56]. Another non-RCT found that the incidence of adverse events in pediatric patients using topical NSAIDs was 14/104, and the incidence of treatment-related adverse events was 9/104. No serious adverse events occurred [134] (Supplemental Table S12).

##### Geriatric Patients

A subgroup analysis of five RCTs [135,136,137] (538 patients) with knee or hip arthritis suggested that compared to no treatment, topical NSAIDs increased the risk of treatment-related adverse events in elderly patients (RR 2.89, 95% CI 1.49–5.60), which was mainly due to an increased risk of local adverse reactions (RR 7.51, 95% CI 3.17–17.78). However, topical NSAIDs did not significantly increase gastrointestinal or cardiovascular adverse events (Supplemental Table S12). Another study showed that the older patients receiving topical NSAIDs reported up to 39.3% application site adverse events and up to 17.5% systemic adverse events [122].

##### Patients with Cardiovascular Diseases

Only indirect evidence comparing cardiovascular adverse events was found in patients with rheumatoid arthritis treated with topical and oral NSAIDs in a Taiwanese healthcare database. Topical NSAIDs were associated with a lower risk of cardiovascular events than oral NSAIDs (RR 0.64, 95% CI 0.43–0.95) [138]. In general, selective COX-2 inhibitors have the highest risk of adverse vascular reactions. However, the cardiovascular risk of non-selective NSAIDs is a concern, especially for those with potent COX-2 inhibitory effects (such as diclofenac). The cardiovascular risks of ibuprofen and naproxen are relatively low. Although patients with cardiovascular disease are discouraged from using NSAIDs, when necessary, physicians and patients should thoroughly evaluate the benefits and risks and use NSAIDs with caution [139].

##### Patients with Gastrointestinal Diseases

There is currently no evidence to evaluate the efficacy and safety of topical NSAIDs in treating musculoskeletal pain in patients with gastrointestinal diseases. However, the evidence in Clinical Question 1 suggests that the risk of gastrointestinal adverse events with diclofenac and ketoprofen is comparable to that of a placebo. Compared to oral NSAIDs, topical NSAIDs had a lower risk of gastrointestinal adverse events (RR 0.66, 95% CI 0.56–0.77, *p* < 0.00001) and possibly a lower risk of treatment discontinuation (RR 0.84, 95% CI 0.68 to 1.05, *p* = 0.13) (Supplemental Table S8).

Drug metabolism studies of oral and topical NSAIDs have shown a lower Cmax and a delayed Tmax for topical NSAIDs compared to oral administration [41]. Furthermore, topical NSAIDs are the preferred treatment for high-risk groups in some guidelines because the drug metabolism of topical administration has less impact on systemic exposure [140,141,142].

Rationale: In conclusion, there was limited evidence on using topical NSAIDs in high-risk patient populations. Considering the pharmacokinetic characteristics, topical NSAIDs may be safer than oral NSAIDs. The low or very low quality of evidence suggests that topical NSAIDs are generally safe to use. The guideline panel made recommendations in high-risk populations based on limited evidence and the pharmacokinetic characteristics of topical NSAIDs.

Expert Consensus 12: For patients with musculoskeletal pain during pregnancy, we recommend that women in the third trimester do not use topical NSAIDs. Very low-quality evidence suggests that the short-term use of topical NSAIDs may be safe for women in the second trimester. There is no evidence for topical NSAIDs for women in the first trimester (expert consensus, no evidence).

Expert Consensus 13: For breastfeeding patients with musculoskeletal pain, there is currently no relevant evidence for topical NSAIDs. Therefore, we suggest breastfeeding patients with musculoskeletal pain use topical NSAIDs with caution, start NSAID treatment with higher safety ratings, and monitor adverse events (expert consensus, no evidence).

Expert Consensus 14: Topical NSAIDs are safer than oral NSAIDs due to their lower systemic exposure. Systemic exposure to topical NSAIDs may increase in patients with hepatic insufficiency. We suggest that patients with hepatic insufficiency use topical NSAIDs with caution and monitor adverse events (expert consensus, no evidence).

Expert Consensus 15: Topical NSAIDs are safer than oral NSAIDs due to their lower systemic exposure. Systemic exposure to topical NSAIDs can increase in patients with renal insufficiency. We suggest that patients with renal insufficiency use topical NSAIDs with caution and monitor adverse events (expert consensus, no evidence).

Recommendation 16: We suggest that children with musculoskeletal pain use topical NSAIDs and monitor adverse events (2C).

Recommendation 17: We suggest that older patients with musculoskeletal pain use topical NSAIDs and monitor adverse events (2C).

Expert Consensus 18: There is currently no direct evidence of using topical NSAIDs in patients with musculoskeletal pain who have cardiovascular disease.

Recommendation 19: For patients with musculoskeletal pain combined with gastrointestinal diseases, we suggest using topical NSAIDs, and adverse events must be monitored (2D).

#### 3.3.6. Clinical Question #6. When Patients Use Topical NSAIDs, Does Pharmacist Intervention (Medication Education, Consultation, etc.) Help Improve Patient Adherence and Increase Efficacy?

After comprehensive evidence retrieval and screening, only three non-RCTs were included as indirect evidence. At the same time, expert experience in clinical practice was collected through online questionnaires to form guiding opinions and standardize clinical practice. Indirect evidence shows that pharmacist intervention can improve the rational use of analgesic drugs, improve short-term pain relief, and help identify and manage adverse events in different settings, such as primary care and clinics [143,144,145]. The findings illustrate the potential value of pharmacist-led pain management.

Expert experience shows that patients receiving pharmacist services are mostly older, with musculoskeletal pain involving multiple joints and other underlying diseases, or taking concomitant medications. Pharmacist services include medication education, prescription review, medication consultation, etc. The participation of dedicated pharmacists in pain management helps optimize medication regimens, reduce adverse effects and medical costs, and improve patient satisfaction. Pharmacy services enhance patient awareness of drugs and medication adherence and avoid wrong medication behaviors. Furthermore, pharmaceutical care helps identify high-risk groups and early signs of severe adverse events, prevent them as soon as possible, and protect patient safety.

Rationale: In conclusion, there was no directly relevant evidence on the pharmacist intervention and service. The guideline panel made the following recommendation based on clinical experience to guide the optimal use of topical NSAIDs and lower the risk of adverse events.

Recommendation 20: We recommend the pharmacist service for patients with musculoskeletal pain who use topical NSAIDs (1C).

## 4. Discussion

These are the first clinical practice guidelines for treating musculoskeletal pain with topical NSAIDs, focusing on drug choice, drug interaction and safety, and pharmacist service. The approach focuses more on issues related to the rational use of topical NSAIDs, and the recommendations are patient-centered. Given the effectiveness and safety of topical NSAIDs and their broad application in musculoskeletal pain, the rational use of topical NSAIDs is critical. We formulated 11 recommendations and nine expert consensuses on the use of topical NSAIDs based on the evidence from the systematic review, patient preferences, and expert experience. Overall, the curative effect of topical NSAIDs in treating musculoskeletal pain is similar to that of oral NSAIDs. However, the risk of systemic adverse events is lower, and the risk of interaction with other drugs is lower, which has certain advantages in patient populations who have comorbidities or who take concomitant medication. Furthermore, pharmacist interventions can play an active role in musculoskeletal pain management.

There are several limitations. First, most recommendations on topical NSAIDs, interactions with other drugs, use in special populations, and pharmacist intervention are based on indirect evidence or expert evidence due to limited clinical evidence. There were only studies with a high risk of bias, which further led to low-quality evidence or not enough evidence to form a recommendation. More RCTs should be conducted to clarify these clinical issues. Second, the outcomes included in the research evaluations often focus only on pain relief and the risk of adverse events. Some critical outcomes, such as treatment satisfaction, ability to return to work, medication adherence, and incremental cost-effectiveness ratio, have not been systematically evaluated. Future RCTs should assess a broader range of outcomes to better understand the full impact of interventions. Third, the focus of experts on the safety of NSAIDs may have influenced the recommendations for the use of topical NSAIDs in special populations. Fourth, the guidelines do not clearly define the exact conditions and characteristics of a special population, such as liver or kidney insufficiency and cardiovascular or gastrointestinal diseases, to maintain a broad representation and increase the applicability of recommendations.

The guideline panel aims to distribute and promote the guideline in various ways to ensure that clinicians, pharmacists, and nurses fully understand and apply the guidelines correctly. We will monitor and evaluate the implementation of the guideline recommendations, the acceptance of the guideline recommendations in clinical practice, and their impact on treatment decisions and patient outcomes. Meanwhile, we will monitor the evidence on the topical NSAIDs, and when possible, the guideline recommendations and the strength of the recommendation will be updated.

## 5. Conclusions

The guidelines summarized the relevant evidence on topical NSAIDs and recommended using topical NSAIDs in treating musculoskeletal pain based on high-quality evidence. Considering the route of administration and the metabolic characteristics of topical NSAIDs, topical NSAIDs may be safer than oral NSAIDs. Using topical NSAIDs in high-risk groups, such as those with comorbidities, or receiving concurrent medications is recommended. Due to the increasing popularity of topical NSAIDs in musculoskeletal pain, the rational use of these drugs deserves attention. The guidelines focus on the most concerning issues for healthcare personnel, including clinicians and pharmacists, and patients with musculoskeletal pain when using topical NSAIDs. The guideline development process was rigorous. These are the first standard guidelines for treating musculoskeletal pain with topical NSAIDs, focusing on drug selection, drug interaction, and safety from a pharmaceutical perspective. Therefore, the guidelines can support decision making for healthcare personnel and patients and facilitate the rational use of topical NSAIDs in clinical practice. The guideline panel will continuously monitor the relevant evidence on topical NSAIDs and update pertinent recommendations in due course.

## Figures and Tables

**Table 1:** Grading of GRADE recommendation strength.

Recommendation Strength	Code	Balance of Benefits and Harms	Applicable Patient Population
Strong	1	The evaluators were convinced that the benefits of the intervention outweighed the harms and vice versa.	Generally applicable to patients in most cases.
Weak	2	The pros and cons were uncertain or the evidence, regardless of quality, showed comparable pros and cons.	Applicable to many patients, but may vary depending on circumstances or patient values and preferences.
Expert consensus	3	There was no relevant evidence to prove the benefits and harms.	The choice needs to be made on a case-by-case basis or based on the patient’s values and preferences.

## Data Availability

Not applicable.

## Supplementary Materials

The following supporting information can be downloaded at: https://www.mdpi.com/article/10.3390/jcm12041544/s1.
