# Supplementary material for: Multidisciplinary Guidelines for the Rational Use of Topical Non-Steroidal Anti-Inflammatory Drugs for Musculoskeletal Pain (2022)
Source: J Clin Med. 2023 Feb 15;12(4):1544. doi: 10.3390/jcm12041544 (PMC9966446; doi:10.3390/jcm12041544)
Supplement: Supplementary file 1 [file jcm-12-01544-s001.zip › jcm-2132168-supplementary.pdf]

Supplemental Tables and Figures

Supplementary Table S1. The composition and responsibilities of the guideline working groups

Group	Members	Responsibility
Steering Group（6 people）	Pharmacy (3), clinical medicine (2), guideline development methodology expert (1)	(1) Determine the scope of the guideline; (2) Set up a guideline development group, secretary group, and external review team; (3) Preliminarily construct PICO questions; (4) Review the declaration of conflict of interest form and supervise the guideline formulation process; (5) Review and approve the recommendations and publication of this guideline.
Development group (25 people)	Clinical pharmacy (13), evidence-based pharmacy (1), pharmacoeconomics (1), pharmaceutics (1), orthopedic surgery (3), rheumatology and immunology (3), pain specialist (2), guideline development methodology expert (1)	(1) Determine the inclusion and ranking of clinical problems and outcome indicators; (2) Guide the secretary group to complete the survey of patient preferences and values; (3) Guide and assist the systematic review team in completing evidence retrieval and evaluation; (4) Form recommendations; (5) Complete the writing and finalization of the full text of this guideline.
External review team（2-3 people）	Stakeholders not directly involved in the development of this guideline, including medical workers, patients or their families, etc.	Review draft guidelines and make comments and suggestions.
Secretary Group（19 people）	Researchers with extensive experience in guideline development	(1) Record the formulation process of this guideline in detail; (2) Coordinate various matters during the formulation process of this guideline.

Note：PICO (P- Patient; I- Intervention; C- Control; O- Outcome).

Supplemental Table S2. Clinical questions and outcomes included in this guideline

1 Clinical Question
1.1 Should patients with musculoskeletal pain use topical NSAIDs for pain relief? The following subgroups should be considered:<br>1) Acute musculoskeletal pain (including strain type, sprain type, and overuse type)<br>2) Chronic musculoskeletal pain (including hand arthritis, hip arthritis, knee arthritis, ankle arthritis, polyarthritis, chronic low back pain, and chronic post-traumatic limb pain)<br>*1.2 What topical NSAIDs should be recommended to relieve pain in patients with musculoskeletal pain? The following subgroups should be considered:*<br>1) Are there differences in the efficacy of topical NSAIDs in different dosage forms?<br>2) Are there differences in the efficacy of topical NSAIDs with different ingredients?<br>*1.3 Should fixed-dose or on-demand topical NSAIDs be used for patients with musculoskeletal pain?*<br>*1.4 When patients with musculoskeletal pain use topical NSAIDs, should clinicians be concerned about drug interactions with the other drugs? The following subgroups should be considered:*<br>1) oral NSAIDs<br>2) oral acetaminophen<br>3) warfarin (for the risk of bleeding and monitoring the INR value)<br>4) ACEI, ARB, and beta-blocker<br>*1.5 Can patient populations with high risk of adverse events use topical NSAIDs? The following subgroups should be considered:*<br>1) Pregnant and lactating patients<br>2) Patients with hepatic insufficiency<br>3) Patients with renal insufficiency<br>4) Pediatric patients<br>5) Elderly patients<br>6) Patients with cardiovascular disease<br>7) Patients with gastrointestinal diseases<br>*1.6 When patients use topical NSAIDs, does pharmacist intervention (medication education, consultation, etc.) help improve patient adherence and increase efficacy?*
2 Effectiveness outcomes<br>2.1 Degree of pain relief<br>2.2 WOMAC score<br>2.3 NNT<br>2.4 Degree of improvement in body function (range of motion)<br>2.5 Degree of symptom improvement (swelling)<br>2.6 Joint stiffness<br>2.7 Quality of life score<br>2.8 Treatment satisfaction<br>2.9 Restoration of work capacity<br>2.10 Proportion of patients with pain relief<br>2.11 Incidence of musculoskeletal injury<br>2.12 Length of hospital stay<br>2.13 Readmission rate
3 Safety outcomes<br>3.1 Gastrointestinal toxicity<br>3.2 Cardiovascular toxicity<br>3.3 Skin Toxicity<br>3.4 Organ damage<br>3.5 Discontinuation rate due to adverse events
4 Medication adherence outcomes<br>4.1 Medication adherence
5 Economic outcomes

5.1 Minimum cost
5.2 Cost-effectiveness ratio
5.3 Incremental cost-effectiveness ratio
5.4 Incremental cost-utility ratio

Note: NSAIDs, non-steroidal anti-inflammatory drugs; INR, international normalized ratio; ACEI, angiotensin-converting enzyme inhibitors; ARB, Angiotensin Receptor Blocker; WOMAC, Western Ontario and McMaster Universities Osteoarthritis Index; NNT, number needed to treat.

Supplemental Table S3. Risk of bias assessment tools for different types of studies

Study Type	Quality Assessment Tool
Clinical practice guidelines (including expert consensus), systematic review, meta-analysis, network meta-analysis	Appraisal of Guidelines for Research & Evaluation Instrument (AGREE II), A Measurement Tool to Assess Systematic Reviews (AMSTAR 2), Risk of Bias in Systematic Review (ROBIS)
Randomized controlled trial (RCT)	Cochrane risk of bias tool (ROB)
Observation study	Risk Of Bias In Non-randomised Studies - of Interventions (ROBINS-I)
Pharmacoeconomics study	Consolidated Health Economic Evaluation Reporting Standards, (CHEERS) Statements
Case report research	Consensus-based Clinical Case Reporting Guideline (CARE)

Supplemental Table S4. GRADE evidence quality grading

Grade	Definition
High (A)	We are very confident that the true effect lies close to that of the estimate of the effect.
Moderate (B)	We are moderately confident in the effect estimate: the true effect is likely to be close to the estimate of the effect, but there is a possibility that it is substantially different
Low (C)	Our confidence in the effect estimate is limited: the true effect may be substantially different from the estimate of the effect.
Very low (D)	We have very little confidence in the effect estimate: the true effect is likely to be substantially different from the estimate of effect

Note: GRADE, grading of recommendations assessment, development and evaluation.

Supplemental Table S5. Characteristics of marketed topical NSAIDs

Formulation	Generic Name	Trade Name	Specification	Dosage
Plaster	Ibuprofen	Nurofen 24-Stunden Schmerzpflaster	200 mg	1 patch each time, once a day
	Diclofenac Sodium	Voltarol 140 mg Medicated Plaster	140 mg	1 patch each time, once in the morning and evening, the maximum daily dose is 2 patches
Gel	Piroxicam	Feldene Gel	0.50%	Apply 1g (about 3 cm or 1/4 inch) to the affected area each time, 3 to 4 times a day
	Ibuprofen	Ibugel	5%	Apply appropriate amount to the affected are
	Ibuprofen	Ibugel Forte	10%	Apply 2-5 cm (50-125 mg ibuprofen) to the affected area each time, up to 3 times a day
	Ibuprofen	Burana	5%	Apply 4-10 cm (100-250 mg ibuprofen) to the affected area each time, up to 3 times a day, the maximum daily dose is 15 g gel, equivalent to 750 mg ibuprofe
	Ibuprofen-peppermint	Duo-Kraft	Ibuprofen 5.0% w/w; L- Menthol 3.0% w/w	1-4 cm each time, apply to the affected area, 2-3 times a day, and the interval between each application should not be less than 4 hours
	loxoprofen sodium	Loxonin Gel	1%	Apply appropriate amount to the affected area, several times a day
	Niflumic Acid	Niflugel	2.5%	2.5 cm (1g) each time, 2 to 4 times a day
	Diclofenac diethylamine	Diclofenac Sigillata Schmerzgel	20mg/g	1-4 g gel (equivalent to cherry to walnut size, 20-80 mg diclofenac sodium) each time, the maximum daily dose is 8 g gel (equivalent to 160 mg diclofenac sodium)
	Diclofenac	Voltaren	200mg/20g	Apply an appropriate amount according to the size of the sore

	diethylamine			area, and rub gently to make the product penetrate the skin, 3 to 4 times a day
	Diclofenac Sodium	Voltaren Gel	1%	Lower limbs: each joint 4 g, 4 times a day, any single joint of the lower limbs should not exceed 16g/day; upper limbs: each joint 2 g, 4 times a day, the dosage of any single joint of the upper limbs should not exceed 8 g/day; daily maximum dose: not to exceed 32 g daily in all affected joints
	Ketoprofen	Oauvail Gel/Sector Gel Fastum	2.5%	2-4 g (5-10 cm) each time, 2-4 times a day, usually the maximum dose is 15g per day
	Indomethacin	IDOMETHINE KOWA GEL	1%	Apply appropriate amount to the affected area, several times a day
	Felbinac	通尔其	10g: 0.3g; 20g: 0.6g	1g each time, 2 to 4 times a day. If there are multiple damages, the total amount per day should not exceed 25g (equivalent to 25 times)
Cataplasms	Flurbiprofen	Zepolas Pap	40, 80 mg	2 times a day, apply to the affected area
	loxoprofen sodium	Loxoprofen Na Pap	100, 200 mg	One time a day, apply to the affected area
	Ketoprofen	Mohrus Pap	30 mg, 60 mg	2 times a day, apply to the affected area
	Ketoprofen	Mohrus Pap	120, 240mg	One time a day, apply to the affected area
	Indomethacin	Catlep, KOWA	35, 70mg	1-2 times a day, apply to the affected area
Foaming agent	Ibuprofen	Ibumousse	5% w/w	1-2 g each time (1-2 golf ball size), 3-4 times a day
	Felbinac	Traxam	3.17% w/w	4 cm each time (1g, the size of a golf ball), 2 to 4 times a day, the total daily dose should not exceed 25g
Spray	Ibuprofen	Buleve Pain Relief 5% Spray	5%	5-10 presses (1-2ml) each time, 3-4 times a day, with an interval of at least 4 hours between administrations, and no more than 40 presses (8ml) within 24 hours

	loxoprofen sodium	Loxoprofen Na Spray	1%	Spray an appropriate amount on the affected area several times a day
	Diclofenac Sodium	Voltarol®Active 4% Cutaneous Spray	4%	4-5 presses each time (0.8-1.0 g contains 32-40 mg diclofenac sodium), the maximum single dose is 1.0g three times a day, and the maximum daily dose is 15 presses (3.0g contains 120mg diclofenac sodium)
	Diclofenac Sodium	Difene Spray Gel	4%	4-5 presses each time, the maximum single dose is 5 presses 3 times a day, and the maximum daily dose is 15 presses
Aerosol	Diclofenac Sodium	劲通，好德快	1.25%	Spray on the affected area, the time of spraying should not exceed 2 seconds (spray about 25mg), 3 times a day, the total daily dosage should not exceed 150mg
Solution	Diclofenac Sodium	Pennsaid	2% w/w	2 presses (40 mg) on each knee, 2 times a day
	Diclofenac Sodium	DICLOFENAC SODIUM 1.5%	1.5% w/w	40 drops/time on each knee, 4 times a day
	Ketoprofen	Sector Lotion	3%	Apply appropriate amount to the affected area, several times a day
	Indomethacin	Inteban	1%, 1.5%	Apply appropriate amount to the affected area, 2-3 times a day
	Felbinac	全力	10ml: 0.3g	1 mL each time, 2 to 4 times a day. If there are multiple damages, the total amount of this product should not exceed 25g per day (equivalent to 25 times)
Cream	Ibuprofen	Vesicum	5%	50-125 mg each time (4-10 cm), 3-4 times a day
	Ketoprofen	Sector Cream	3%	Apply appropriate amount to the affected area, several times a day
	Indomethacin	KOWA	1%	Apply appropriate amount to the affected area, several times a day

Ointment	Piroxicam	Baxo	0.50%	Apply appropriate amount to the affected area, several times a day
	Indomethacin	Inteban	1%	Apply appropriate amount to the affected area, several times a day
Patch	Piroxicam	亮克	14mg	Stick on the affected area, 1 patch each time, once a day
	Piroxicam	Trast	48mg	Stick on the affected area, 1 patch each time, once every 2 days. 1 patch daily when bathing, showering or sweating
	Flurbiprofen	Zepolas	20, 40mg	2 times a day, apply to the affected area
	loxoprofen sodium	Loxonin	50, 100mg	1 time a day, apply to the affected area
	Diclofenac Eporamide	Flector	1.30%	1 patch (180 mg) each time, 2 times a day
	Ketoprofen	Mohrus Tape	20, 40mg	1 time a day, apply to the affected area
	Indomethacin	Catlep	35mg	2 times a day, apply to the affected area

Supplemental Table S6. Comparison of the efficacy and adverse reactions of topical NSAIDs and placebo in the treatment of acute musculoskeletal pain –Evidence Summary

Outcomes	Anticipated absolute effects* (95% CI)		Relative effect (95% CI)	Number of participants (studies)	Certainty of the evidence (GRADE)
	Risk with topical NSAIDs	Risk with placebo			
Treatment Success	660 per 1000	426 per 1000	1.55 (1.48, 1.64)	5539 (35 trials)	High
Local adverse events	25 per 1000	25 per 1000	1.01 (0.84, 1.21)	7642 (42 trials)	High

Note: *The risk in the intervention group (and its 95% confidence interval) is based on the assumed risk in the comparison group and the relative effect of the intervention (and its 95% CI). CI: Confidence interval; MD: Mean difference.

Supplemental Table S7. Comparison of the efficacy and adverse reactions of topical NSAIDs and placebo in the treatment of chronic musculoskeletal pain - Evidence Summary

Outcomes	Anticipated absolute effects* (95% CI)		Relative effect (95% CI)	Number of participants (studies)	Certainty of the evidence (GRADE)
	Risk with topical NSAIDs	Risk with placebo			
Treatment Success					
Diclofenac（> 6weeks）	547 per 1000	456 per 1000	1.20 (1.12, 1.29)	2343 (5 trials)	MODERATE
Ketoprofen	497 per 1000	456 per 1000	1.09 (1.01, 1.17)	2573 (4 trials)	MODERATE
Local adverse events					
Diclofenac	129 per 1000	114 per 1000	1.13 (1.00, 1.29)	4452 (18 trials)	MODERATE
Ketoprofen	118 per 1000	114 per 1000	1.04 (0.85, 1.27)	2621 (4 trials)	MODERATE
Gastrointestinal adverse events					
Diclofenac	42 per 1000	37 per 1000	1.13 (0.82, 1.55)	3398 (14 trials)	MODERATE
Ketoprofen	36 per 1000	37 per 1000	0.96 (0.69, 1.32)	2621 (4 trials)	MODERATE
Discontinuation due to adverse reactions					
Diclofenac	47 per 1000	32 per 1000	1.46 (1.10, 1.94)	4117 (17 trials)	MODERATE

Outcomes	Anticipated absolute effects* (95% CI)		Relative effect (95% CI)	Number of participants (studies)	Certainty of the evidence (GRADE)
	Risk with topical NSAIDs	Risk with placebo			
Ketoprofen	41 per 1000	32 per 1000	1.28 (0.92, 1.78)	2621 (4 trials)	MODERATE

Note: *The risk in the intervention group (and its 95% confidence interval) is based on the assumed risk in the comparison group and the relative effect of the intervention (and its 95% CI). CI: Confidence interval; MD: Mean difference.

Supplemental Table S8. Comparison of the efficacy and safety of topical versus oral NSAIDs for the treatment of chronic musculoskeletal pain - Evidence Summary

Outcomes	**Anticipated absolute effects*** (95% CI)		Relative effect (95% CI)	Number of participants (studies)	Certainty of the evidence (GRADE)
	Risk with topical NSAIDs	Risk with oral NSAIDs			
Treatment success	607 per 1000	583 per 1000	1.04 (0.97, 1.12)	2197 (9 trials)	MODERATE
Local adverse events	114 per 1000	34 per 1000	3.36 (2.53, 4.45)	2120 (9 trials)	MODERATE
Gastrointestinal adverse events	89 per 1000	135 per 1000	0.66 (0.56, 0.77)	2430 (10 trials)	MODERATE
Drug discontinuation due to adverse events	40 per 1000	47 per 1000	0.84 (0.68, 1.05)	2431 (10 trials)	MODERATE
Drug discontinuation due to suboptimal treatment	64 per 1000	32 per 1000	1.98 (1.25, 3.12)	1667 (7 trials)	MODERATE

Note: *The risk in the intervention group (and its 95% confidence interval) is based on the assumed risk in the comparison group and the relative effect of the intervention (and its 95% CI). CI: Confidence interval; MD: Mean difference.

Supplemental Table S9. Comparison of the efficacy of topical NSAIDs with different components for musculoskeletal pain - Evidence Summary

NSAIDs	Number of studies	Number of study participants	Effect estimates Relative Risk (95% CI)	NNT (95% CI)	Evidence quality（GRADE）
Diclofenac	13	2964	1.55 (1.44, 1.66）	4.27（3.56, 5.33）	Moderate [a]
Ibuprofen	7	698	1.84 (1.58, 2.14)	2.79（2.06, 4.05）	Moderate [a]
Ketoprofen	7	683	1.56 (1.37, 1.77)	4.19（3.05, 6.34）	Moderate [a]
Piroxicam	4	504	1.48 (1.27, 1.73)	4.89（3.22, 8.69）	Moderate [a]
Indomethacin	3	341	1.26 (1.03, 1.55)	9.03（4.27, 78.25）	Low [a, b]
Benzydamine	3	193	1.15 (0.96, 1.38)	15.65（6.18，-）	Low [a, b]
Etofenamate	1	156	6.83 (3.08, 15.16)	0.40（0.17, 1.13）	Low [b, c]

Note: NSAIDs, nonsteroidal anti-inflammatory drugs; CI, confidence interval; NNT, number needed to treat; GRADE, grading of recommendations assessment, development and evaluation. a. risk of bias; b. imprecision; c. indirectness.

Supplemental Table S10. Comparison of the efficacy and safety of different topical NSAIDs for the treatment of hip/knee arthritis

Topical NSAIDs	Effect Size for Pain Relief (95% CI)	Probability of achieving minimal clinically meaningful difference	Odds ratio for dropout due to adverse events (95% CI)
Diclofenac (70-81 mg)	−0.54 (−0.77, −0.31)	92.3	1.14 (0.74, 1.72)
Diclofenac (140-160 mg)	−0.61 (−0.87, −0.35)	96.3	1.58 (0.77, 3.34)
Flurbiprofen patch (≤20 mg)	−0.25 (−0.92, 0.42)	36.2	0.40 (0.03, 4.14)
Flurbiprofen patch (40 mg)	−0.41 (−1.20, 0.37)	53.9	0.32 (0.01, 3.82)
Ibuprofen (1500 mg)	−0.19 (−1.03, 0.66)	33.7	—
Ketoprofen (50 mg)	−0.15 (−0.64, 0.33)	18.7	0.67 (0.25, 1.65)
Ketoprofen (100 mg)	−0.22 (−0.49, 0.06)	14.1	0.99 (0.44, 2.15)
Ketoprofen (200-220 mg)	−0.23 (−0.39, −0.06)	4.4	1.27 (0.79, 2.02)
Piroxicam (15 mg)	0.39 (−0.49, 1.25)	4.3	1.32 (0.31, 5.74)

Note: CI, confidence interval; NSAIDs, nonsteroidal anti-inflammatory drugs.

Supplemental Table S11. Evaluation of drug interactions between topical NSAIDs and other drugs - Evidence Summary

Outcomes	Anticipated absolute effects* (95% CI)		Relative effect (95% CI)	Number of participants (studies)	Certainty of the evidence (GRADE)	Comments
	Risk with potential drug-drug interaction	Risk with no potential drug-drug interaction				
Potentiation of warfarin	5 cases	-	-	12<br>(1 case series, 1 case report)	Very low	Only 1 trial, small sample size; very serious risk of bias due to lack of control, very serious imprecision, and very serious indirectness
Risk of Adverse Reactions	At least one AE was experienced by 62.6% (107/171) of patients with at least 1 drug-drug interaction risk<br>Gastrointestinal: 5.3% (9/171)<br>Cardiovascular: 4.7% (8/171)<br>Renal: 1.2% (2/171)<br>Hepatic: 0%	At least one AE was experienced by 55.4% (46/83) of patients<br>Gastrointestinal: 7.2% (6/83)<br>Cardiovascular: 1.2% (1/83)<br>Renal: 0%<br>Hepatic: 1.2% (1/83)		254 (1 trial)	Low	Only 1 study, small sample size; imprecision and indirectness

Note: *The risk in the intervention group (and its 95% confidence interval) is based on the assumed risk in the comparison group and the relative effect of the intervention (and its 95% CI). CI: Confidence interval; MD: Mean difference.

Supplemental Table S12. Safety of Topical NSAIDs in Special Populations - Evidence Summary

Outcomes	Anticipated absolute effects* (95% CI)		Relative effect (95% CI)	Number of participants (studies)	Certainty of the evidence (GRADE)	Comments
	Risk with topical NSAIDs	Risk with placebo				
Children						
Pain relief	2.5% Ketoprofen gel:<br>20 (13–28) at 15 min<br>35 (29–41) at 30 min	5 (4–10) at 15 min;<br>9 (6–16) at 30 min	-	112 (1 trial)	Low	Only 1 trial, small sample size; imprecision and indirectness
Adverse events	0	0	Not estimable	112 (1 trial)	-	-
Risk of adverse events	Adverse events: 14/104<br>Possibly treatment-related events: 9/104<br>Serious: 0			104<br>(1 non randomized study)	Low	Only 1 study, small sample size; imprecision and indirectness

Older adults

Outcomes	Anticipated absolute effects* (95% CI)		Relative effect (95% CI)	Number of participants (studies)	Certainty of the evidence (GRADE)	Comments
	Risk with topical NSAIDs	Risk with placebo				
Risk of treatment-related adverse reactions	120 per 1000	42 per 1000	2.89 (1.49, 5.60)	538 (5 trials)	Low	Small sample size and number of events; imprecision and indirectness (Diclofenac for knee and hip osteoporosis)
Local adverse reactions	578 per 1000	77 per 1000	7.51 (3.17, 17.78)	715 (6 trials)	Low	Small sample size and number of events; imprecision and indirectness (Diclofenac for hand, knee and hip osteoporosis)
Discontinuation due to adverse reactions	58 per 1000	8 per 1000	7.71 (1.79, 33.20)	538 (5 trials)	Low	Small sample size and number of events; imprecision and indirectness (Diclofenac for knee and hip osteoporosis)
Gastrointestinal adverse reaction	174 per 1000	179 per 1000	0.97 (0.46, 2.02)	715 (6 trials)	Low	Small sample size and number of events; imprecision and indirectness (Diclofenac for hand, knee and hip osteoporosis)

Outcomes	Anticipated absolute effects* (95% CI)		Relative effect (95% CI)	Number of participants (studies)	Certainty of the evidence (GRADE)	Comments
	Risk with topical NSAIDs	Risk with placebo				
Cardiovascular adverse reaction	4 per 1000	0	2.89 (0.12, 70.65)	538 (5 trials)	Very low	Small sample size and number of events; very serious imprecision and indirectness (Diclofenac for knee and hip osteoporosis)

Note: *The risk in the intervention group (and its 95% confidence interval) is based on the assumed risk in the comparison group and the relative effect of the intervention (and its 95% CI). CI: Confidence interval; MD: Mean difference.

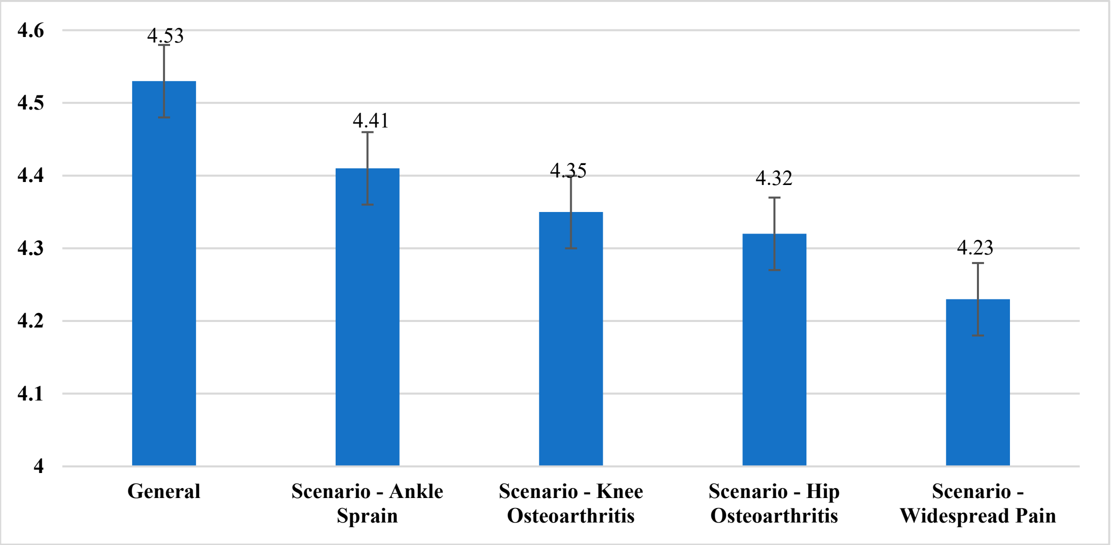

Supplemental Figure S1 Willingness of patients with musculoskeletal pain to accept the use of topical NSAIDs in different scenarios. Note: The degree of patient willingness was assigned using a scale of 1 to 5: very willing = 5, more willing = 4, neutral = 3, less willing = 2, not at all willing = 1.

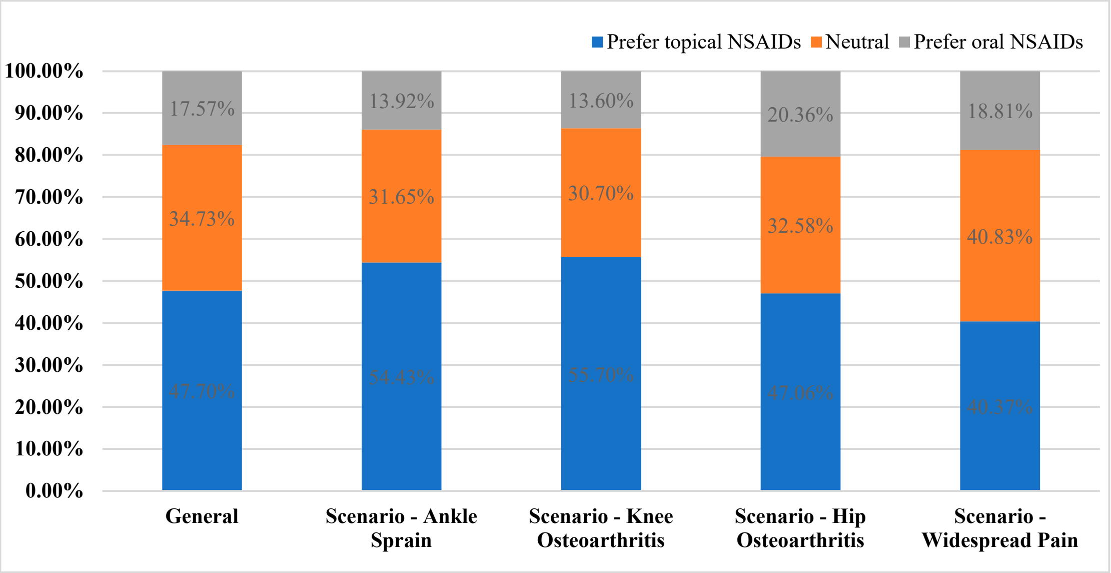

Supplemental Figure S2 Preferences for topical and oral NSAIDs in musculoskeletal pain patients in different contexts. Note: The patient's preference for "topical and oral NSAIDs" was calculated as the topical NSAIDS score - oral NSAIDs score (the result range is - 4 to 4). The larger the value, the more likely the patient is to choose topical NSAIDs, otherwise, the more likely the patient is to choose oral NSAIDs, and 0 means neutral.

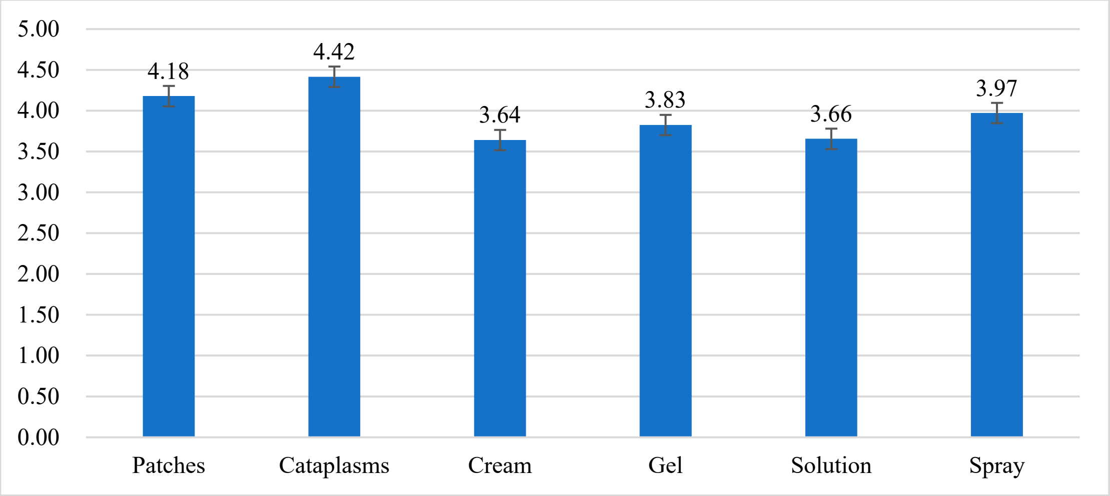

Supplemental Figure S3 Preferences for topical NSAIDs of different dosage in musculoskeletal pain patients. Note: The degree of patient willingness was assigned using a scale of 1 to 5: very willing = 5, more willing = 4, neutral = 3, less willing = 2, not at all willing = 1.
